# Neuroprotective Effects of Black Pepper Cold-Pressed Oil on Scopolamine-Induced Oxidative Stress and Memory Impairment in Rats

**DOI:** 10.3390/antiox10121993

**Published:** 2021-12-15

**Authors:** Nada M. Mostafa, Ahmed M. Mostafa, Mohamed L. Ashour, Sameh S. Elhady

**Affiliations:** 1 Department of Pharmacognosy, Faculty of Pharmacy, Ain-Shams University, Cairo 11566, Egypt; 2 Department of Biochemistry, Faculty of Pharmacy, Ain-Shams University, Cairo 11566, Egypt; ahmedmostafa@pharma.asu.edu.eg; 3 Department of Pharmaceutical Sciences, Pharmacy Program, Batterjee Medical College, Jeddah 21442, Saudi Arabia; 4 Department of Natural Products, Faculty of Pharmacy, King Abdulaziz University, Jeddah 21589, Saudi Arabia; ssahmed@kau.edu.sa

**Keywords:** black pepper, cold-pressed oil, GC–MS, neuroprotective, scopolamine

## Abstract

Oxidative stress is usually associated with many neurodegenerative diseases. In this study, the gas chromatography–mass spectrometry (GC–MS) analysis of cold-pressed oil (CPO) from black pepper (*Piper nigrum*) fruits was performed and its neuroprotective effects were evaluated for the first time. The analysis of CPO revealed the presence of the lignan sesamin (39.78%), the alkaloid piperine (33.79%), the monoterpene hydrocarbons 3-carene (9.53%) and limonene (6.23%), and the sesquiterpene β-caryophyllene (10.67%). Black pepper hydrodistilled oil (HDO) was also comparatively analyzed by GC–MS to show the impact of oil isolation by two different methodologies on their components and class of compounds identified. HDO analysis revealed 35 compounds (99.64% of the total peak areas) mainly composed of monoterpene hydrocarbons (77.28%), such as limonene (26.50%), sabinene (21.36%), and β-pinene (15.53%), and sesquiterpene hydrocarbons (20.59%) represented mainly by β-caryophyllene (19.12%). Due to the low yield obtained for HDO (0.01% *v/w*), only CPO was chosen for the evaluation of its neuroprotective potential. Alzheimer-type dementia was induced in rats by scopolamine intraperitoneal injection (1.5 mg/kg/day) for seven days. CPO was administered orally (100 mg/kg) for a week before scopolamine administration and then concomitantly for another week. Donepezil (1 mg/kg, orally) was used as a reference drug. CPO administration significantly improved the rat behaviors as evaluated by the Morris water maze test, evident from prolongation in time spent in the platform quadrant (262.9%, compared to scopolamine) and increasing in the crossing time by 18.18% compared to the control group. The rat behavior tested by passive avoidance, showed prolongation in the step-through latency compared to control. Moreover, CPO significantly (*p* < 0.05) ameliorated the activities of antioxidant enzymes such as catalase, superoxide dismutase (SOD) and reduced malondialdehyde (MDA) equivalents by 22.48%, 45.41%, and 86.61%, respectively, compared to scopolamine. Furthermore, CPO administration decreased scopolamine-induced elevated acetylcholinesterase levels in rats’ hippocampi by 51.30%. These results were supported by histopathological and in silico molecular docking studies. Black pepper oil may be a potential antioxidant and neuroprotective supplement.

## 1. Introduction

Alzheimer’s is a progressive disease with estimated prevalence of about 47 million individuals worldwide. It is characterized by neurodegeneration of the brain tissues, impairment of memory capabilities, decreased levels of acetylcholine and disturbance of behavior [1,2,3,4]. The antimuscarinic drug scopolamine has been used to induce Alzheimer-type dementia in various animal models by acting as a cholinergic-receptor antagonist [5]. Treatment protocols usually involve cholinesterase inhibitors, such as donepezil, galantamine, and rivastigmine, for mild to moderate cases, while patients demonstrating much severe cases can be also administered *N*-methyl-D-aspartate receptor antagonists, such as memantine. The earlier the diagnosis, the more proper management of Alzheimer’s disease, and the better patient’s quality of life [4]. 

Many AChE inhibitors are used for symptomatic treatment of Alzheimer’s. Unfortunately, these inhibitors are non-selective and demonstrate cholinergic and hepatotoxic adverse effects. As a result, there is an increased need and global attention to the search for a safer management through herbal supplements and natural phytochemicals [6,7]. Additionally, the fear of multiple medications often encountered in elderly patients, as well as other common challenges accompanying the management of old-age dementia, could be reduced or preempted by replacing the drugs with a natural, safe, and edible alternative.

The successful use of natural products for Alzheimer’s management has been previously reported. Different classes of phytochemicals such as flavonoids, tannins, alkaloids, and terpenes exert promising neuroprotective activities via their antioxidant, anti-inflammatory, anti-amyloid, and cholinesterase inhibitory effects [8]. In addition, many edible plants extracts and fractions such as *Curcuma longa*, *Ginkgo biloba*, *Zingiber officinalis*, *Allium sativa*, and *Bombax ceiba* show potential therapeutic activities [9,10].

Black pepper (*Piper nigrum*, Piperaceae) is a climbing vine widely cultivated in tropical regions. It has been used traditionally in colds, flu, fever, arthritic complaints, muscle pain, and gastric disorders. In addition, it is widely used worldwide as a condiment in nearly all savory dishes [11,12]. Various pharmacological activities have been reported for black pepper, such as antimicrobial [13], antioxidant [14], antimutagenic [15], anxiolytic, and antidepressant [11] effects. Chemical investigations have revealed the presence of alkaloids, mainly piperine; carbohydrates; volatile components such as terpenes; proteins; minerals, such as calcium and potassium; and nitrogen-containing compounds [16]. The presence of piperine may be associated, at least in part, with the observed antioxidant activity of black pepper. Piperine has been previously reported to demonstrate in vitro antioxidant potential, free radical scavenging capabilities, and lipid peroxidation inhibition (IC_50_ = 1.23 mM) [17]. Moreover, in vivo studies in rats further validated its antioxidative effect through the evaluation of antioxidant enzymes, such as reduced glutathione, superoxide dismutase, and catalase [14].

The aim of the present study was to evaluate of the neuroprotective effects of black pepper cold-pressed oil (CPO) against scopolamine-induced oxidative stress and memory impairments in rats, together with identifying its bioactive components via GC–MS analysis for the first time. The effect of heat treatment was also studied through comparatively analysis of the hydrodistilled oil composition. The neuroprotective activity was compared to the reference drug donepezil and validated by neurochemical testing, behavioral evaluation, histopathological examination of the rats’ brain tissue, and an in silico molecular docking study.

## 2. Materials and Methods

### 2.1. Plant Material and Chemicals

Black pepper (*Piper nigrum* Wall., family Piperaceae) fruits were obtained from a local market. The fruits were packed, stored at room temperature, and used before the expiry date. Mrs. Treze Labib, Consultant of Plant Taxonomy at the Egyptian Ministry of Agriculture, kindly authenticated the samples. A voucher specimen (PHG-P-PN-1) was kept at the herbarium of the Pharmacognosy Department, Faculty of Pharmacy, Ain Shams University, Cairo, Egypt. Scopolamine was purchased from Sigma Aldrich, St. Louis, MO, USA. Acetylcholinesterase, catalase, and malondialdehyde kits were obtained from Bio-Diagnostics Company, Giza, Egypt. Donepezil tablets were purchased from a local pharmacy. All other chemicals used in the current study were of high analytical grade. 

### 2.2. Clevenger Apparatus, Hydrodistilled and Cold-Pressed Oil 

Clevenger apparatus was purchased from a local supplier (El Borog for Chemicals and glassware, Cairo, Egypt), the capacity of the receiving tube was 25 mL. Dried entire black pepper fruits (150 g) were subjected to hydrodistillation in the Clevenger apparatus as per the Egyptian Pharmacopoeia [18] and other reported methodologies [19,20]. The temperature of the heating mantle was adjusted to 100 °C; then, when water started to boil, the temperature was brought down to 60 °C for four consecutive hours calculated from the beginning of water boiling. After the water was discarded, the obtained oil was collected, dried over anhydrous sodium sulfate, and kept in a sealed vial in the fridge until subsequent GC–MS analysis. The cold-pressed oil was obtained from El Hawag Factory, Badr City, Egypt, under the approval of the Egyptian Ministry of Health (License no. 150/2/159 for the year 2009).

### 2.3. GC–MS Analysis

The chromatographic separation was recorded using Shimadzu GC/MS-QP2010 (Japan) apparatus equipped with Rtx-5MS (Restek, Bellefonte, PA, USA) fused-bonded column (30 mL, 0.25 mm i.d., and 0.25 µm film thickness) that is coupled to SSQ 7000 quadrupole mass spectrometer, Thermo-Finnigan, Germany. The chromatographic procedure and conditions followed the method adopted by Mostafa (2018) [21]. Identification of compounds was performed according to comparing the mass spectra, calculated retention indices by those published in Adams [22], NIST Mass Spectral Library, co-chromatographic standards, and other literature [20,23,24].

### 2.4. Experimental Animals

Sprague Dawley male rats (150–200 g) were purchased from National Research Center, Giza, Egypt. They were acclimatized in the animal house facility, Faculty of Pharmacy, Ain Shams University, for at least a week before experimenting. The rats were supplied with standard food pellets, kept at 23 ± 2 °C, and had access to water ad libitum. Their treatment protocol was approved (approval number: 31 July 2018) by the Ethical Committee for Animal Use, Faculty of Pharmacy, Ain Shams University, Cairo, Egypt.

### 2.5. Experimental Design

Animals were divided into four groups; each group consisted of six rats. Group I: Control group received distilled water containing 10% Tween 80 orally [25] for one week and then concomitantly with intraperitoneal injection of normal saline for another week. Group II: received distilled water containing 10% Tween 80 orally a week prior to scopolamine, followed by concomitant administration with scopolamine (1.5 mg/kg/day, intraperitoneally) for another week. The scopolamine was dissolved in normal saline and administered intraperitoneally using an insulin syringe. Group III and IV: received donepezil (1 mg/kg/day) or CPO (100 mg/kg/day, as an emulsion form using distilled water containing 10% Tween 80), respectively, orally a week before scopolamine challenge, then concomitantly with scopolamine (1.5 mg/kg/day, intraperitoneally) [26] for another week.

### 2.6. Morris Water Maze and Passive Avoidance Tests

Both behavioral tests were performed according to the specifications and procedure adopted by Mostafa (2018) [10]. For the Morris water maze test, the time of swimming and the number of crossing times in the quadrant of the removed platform were recorded three times for each rat by a video camera. For the passive avoidance test, the latency times were recorded in the acquisition and retention trials as the time for each rat (up to a maximum of 300 s) to enter the dark chamber.

### 2.7. Rat Brain Sample Preparation and Histopathology

The rats were sacrificed (after being anesthetized) by decapitation after two weeks. For each rat, the skull was opened and the brain was cut along the sagittal plain; three of the resulting half-brains were selected at random from each group of rats and fixed in formalin (10% solution in saline) for histopathology, where they were sectioned parasagittally to access the brain regions shown in Figures 3 and 4 (cf. also [27]), stained with hematoxylin and eosin, and examined by an electric light microscope. The other hemispheres and whole rats’ brains (6 samples from each group) were dissected with the help of pathologist to separate the hippocampus for preparing a 10% *w/v* homogenate in isotonic saline for neurochemical testing. 

### 2.8. Neurochemical Parameters

AChE activity (expressed as nM/min/mg tissue) was tested according to the method adopted by Ellman et al., 1961 [28], while catalase (expressed as U/g tissue), SOD (expressed as U/mg tissue), and MDA (expressed as µmol/g tissue) activities were determined according to the methods of Aebi (1984) [29], Nishikimi et al. (1972) [30], and Ohkawa et al. (1979) [31], respectively. The kits for testing all the neurochemical parameters were purchased from Bio-Diagnostics Company, Giza, Egypt. 

### 2.9. Molecular Docking Study

In silico molecular docking of all the GC–MS-identified constituents of black pepper cold-pressed oil was carried out to the crystal structure of acetylcholinesterase. Docking study was performed using Discovery Studio 4.5 software, Accelrys Inc., San Diego, CA, USA. The crystal structure of acetylcholinesterase was downloaded from the protein data bank (PDB ID 1OCE, 2.70 Å), as co-crystallized with a ligand. Both the enzyme and the compounds (ligands) were prepared at first by the default program protocol. Then, the active binding site of the AChE enzyme was detected via the determination of the binding mode of the co-crystallized reported ligand active conformer. For 3-carene, limonene, β-caryophyllene, piperine, and sesamin, each of the structures were docked into the active binding site pocket of the enzyme by applying the C-Docker protocol. The program supplied the C-Docker binding energies and the 2D- and 3D-binding diagrams for evaluation.

### 2.10. Statistical Analyses

Results were expressed as mean ± SEM, analyzed by one-way ANOVA followed by Tukey post hoc tests; differences were considered statistically significant at *p* < 0.05. Graphs were sketched by GraphPad Prism (version 5.01) software, California, CA, USA.

## 3. Results

Dementia was induced by intraperitoneal injection of 1.5 mg/kg/day scopolamine (dissolved in normal saline) in rats for 1 week. The neuroprotective activity and the memory-enhancing power of CPO (dose; 100 mg/kg, orally) were assessed through comprehensive behavioral, histochemical, and histopathological studies. 

### 3.1. Behavioral Study 

**Figure 1:**
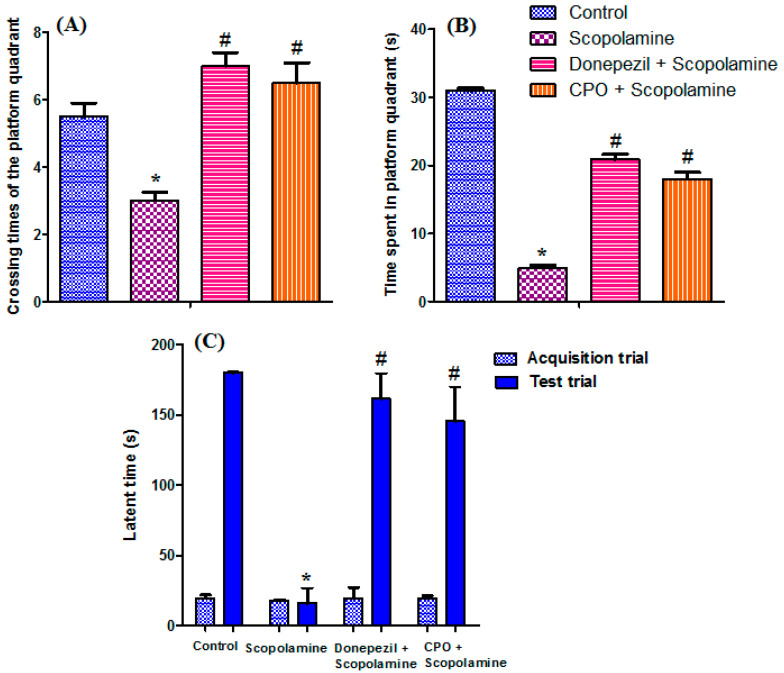
Effect of cold-pressed pepper oil on Morris water maze (**A**,**B**) and passive avoidance tests (**C**). Data are expressed as mean ± SEM, *n* = 6. *, significant difference (*p* < 0.05) from the control group, #, significant difference (*p* < 0.05) from the SC-treated group. This figure was adopted from Mostafa [10].

The rats underwent the Morris water maze test to assess spatial memory and the learning abilities [32]. The protective effect of the oil on scopolamine-induced memory impairment was demonstrated in Figure 1A,B. Prolonged escape latency was observed in the scopolamine-treated group rats, in addition to a marked shortening of time spent in the platform quadrant with a percentage decrease of 84% compared to the control group. Moreover, the dementia-induced group showed a significant decrease in crossing times of the platform quadrant, with a percentage change of −45.45% compared to the control group. However, rats in the CPO-treated group could efficiently finding the quadrant with the platform. They demonstrated a prolongation in the time spent in the platform quadrant with a percentage increase of 262.9% compared to the scopolamine group. They increased crossing times by 18.18% compared to the control group. The memory improvement effects demonstrated by the cold-pressed pepper oil were closely similar and comparable to the reference drug donepezil.

Rat behaviors were also evaluated by performing a passive avoidance test (Figure 1C) to assess long-term memory effects [33], including the acquisition, retention, and retrieval processes. A significant shortening (−91.14%) in the step-through latency was observed in the scopolamine-treated group compared to the control group. Both the oil and the donepezil-treated groups exhibited a prolongation in the step-through latency, with decreases of 19.17 and 10.34%, respectively, compared to the control group. No differences in the latency times were observed among the tested groups in the acquisition trial. 

### 3.2. Neurochemical Study

The evaluation of antioxidant (catalase and MDA equivalents levels) and acetylcholinesterase activity parameters elucidated the potential neurochemical mechanism of memory enhancement demonstrated by the cold-pressed pepper oil. 

**Figure 2:**
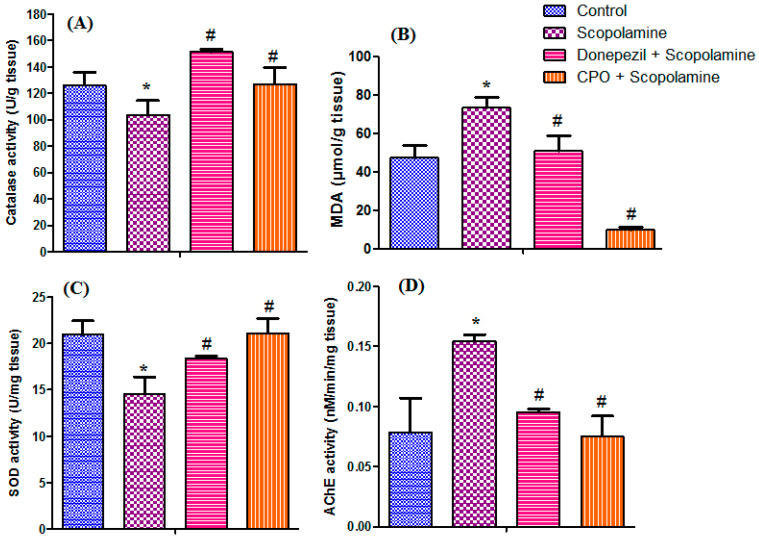
Effect of cold-pressed pepper oil on levels of catalase (**A**), MDA (**B**), SOD (**C**), and AChE (**D**). Data are expressed as mean ± SEM, *n* = 6. *, significant difference (*p* < 0.05) from the control group, #, significant difference (*p* < 0.05) from the SC-treated group. This figure was adopted from Mostafa [10].

The antioxidant activity was evaluated in the brain tissues from rats to reveale the ameliorative effect of the CPO on the oxidative stress status altered by scopolamine (Figure 2A–C). Scopolamine administration decreased catalase activity by 18.25%, along with an increase in the MDA equivalents by 55.27%, compared to the control group. The concomitant administration of CPO or donepezil along with scopolamine resulted in a significant increase (*p* < 0.05) in the scopolamine-induced reduction of catalase activity by 22.48 and 46.04%, respectively, and a related reduction in the MDA equivalents by 86.61 and 31.11%, correspondingly, compared to the scopolamine-treated group. Notably, the CPO treatment significantly reduced MDA equivalents compared to the control group by 79.20%. The donepezil-treated group showed similar results to the control group. Similarly, scopolamine administration decreased the SOD activity levels by 30.62% compared to the control group. The administration of either pepper oil or donepezil along with scopolamine resulted in a significant increase (*p* < 0.05) in the scopolamine-induced reduction of SOD activity, by 45.15 and 26.46%, respectively, compared to groups receiving only scopolamine. It should be noted that treatment with CPO restored SOD levels to those recorded by the control group, with a percentage increase of 0.72% (Figure 2C). Thus, pepper oil demonstrated a potential antioxidant effect through ameliorating the oxidative damage induced by scopolamine, as demonstrated by elevated catalase activity and reduced malondialdehyde levels.

The acetylcholinesterase (AChE) activity levels were determined in the brain tissue of rats to identify the possible mechanism of the neuroprotective effect of CPO. A relevant increase in AChE activity (87.5%) was observed in rats receiving scopolamine alone. Treating rats with either donepezil or CPO resulted in a significant decrease (*p* < 0.05) by 36.67 and 51.30%, respectively, in the elevated levels of AChE. Compared to the control group, the treatment with CPO restored the normal level of AChE, with a decrease of 3.85% (Figure 2D).

### 3.3. Histopathology Studies

**Figure 3:**
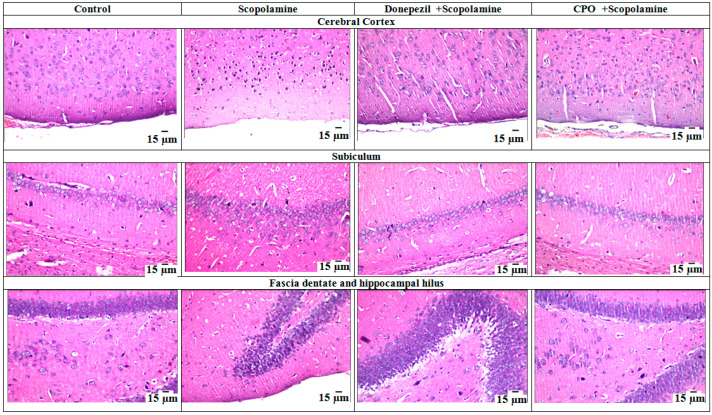
Microphotographs (stained by hematoxylin and eosin) showing cerebral cortex, subiculum, fascia dentate, and hippocampal hilus from the brain of rats in the control, scopolamine-, donepezil-, and pepper oil-treated groups. This figure was adopted from Mostafa [10].

**Figure 4:**
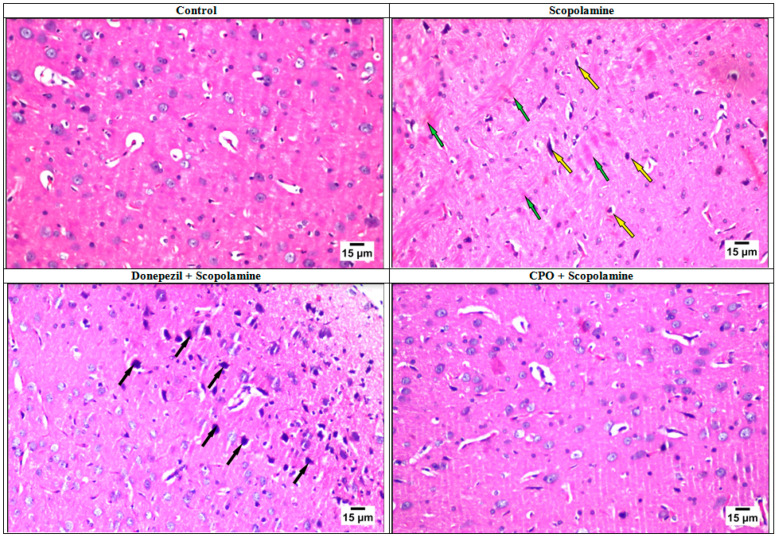
Microphotographs (stained by hematoxylin and eosin) showing striatum in the brain of rats from the control, scopolamine-, donepezil-, and pepper oil-treated groups. Pyknotic and degenerating neurons (black arrows), eosinophilic plaques (green arrows), and neuronal degeneration (yellow arrows). This figure was adopted from Mostafa [10].

Histopathological studies of the brain tissues from the rats supported the above results. No changes in the histopathological structures were observed in control, donepezil-treated, or CPO-treated groups. The normal structure of the different brain regions (cerebral cortex, subiculum, fascia dentate, and hilus of the hippocampus) was recorded (Figure 3). Some nuclear pyknosis and degeneration were observed only in the striatum neurons of the donepezil-treated group (Figure 4). On the contrary, scopolamine administration (1.5 mg/kg/day) intraperitoneally to the rats for a week resulted in alterations in the normal features of the brain tissues. The striatum demonstrated small eosinophilic plaques in addition to neuronal nuclear pyknosis and degeneration (Figure 4).

### 3.4. Chemical Composition of the Oils

Comparative gas chromatographic analyses of the hydrodistilled (HDO) and CPO oils of black pepper (Figures S1 and S2) are compiled in Table 1. The HDO oil was colorless with a characteristic aromatic odor and yield of 0.01% *v/w*, while the CPO was yellow, with a faint odor. Thirty-five compounds, representing 99.64% of the total peak area, were identified in HDO. Monoterpene hydrocarbons (77.28%) represented the primary class of compounds present in the HDO, composed principally of limonene (26.50%), sabinene (21.36%), and β-pinene (15.53%); β-caryophyllene (19.0%) was the most abundant of the sesquiterpene hydrocarbons (20.59%). The CPO revealed the presence of only five compounds, comprising mainly the lignan sesamin (39.78%) and the alkaloid piperine (33.79%). Other components were monoterpene hydrocarbons (15.76%), comprising 3-carene (9.53%) and limonene (6.23%). β-Caryophyllene (10.67%) was the only sesquiterpene detected.

### 3.5. Molecular Docking Study

**Figure 5:**
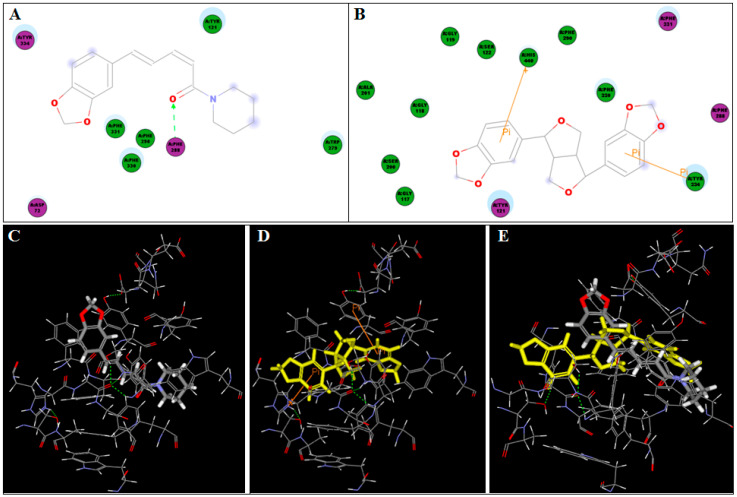
2D- and 3D-binding diagrams of piperine (**A**,**C**), sesamin (**B**,**D**), and the 3D-binding diagram of the two compounds concomitantly (**E**) to AChE crystal structure.

**Figure 6:**
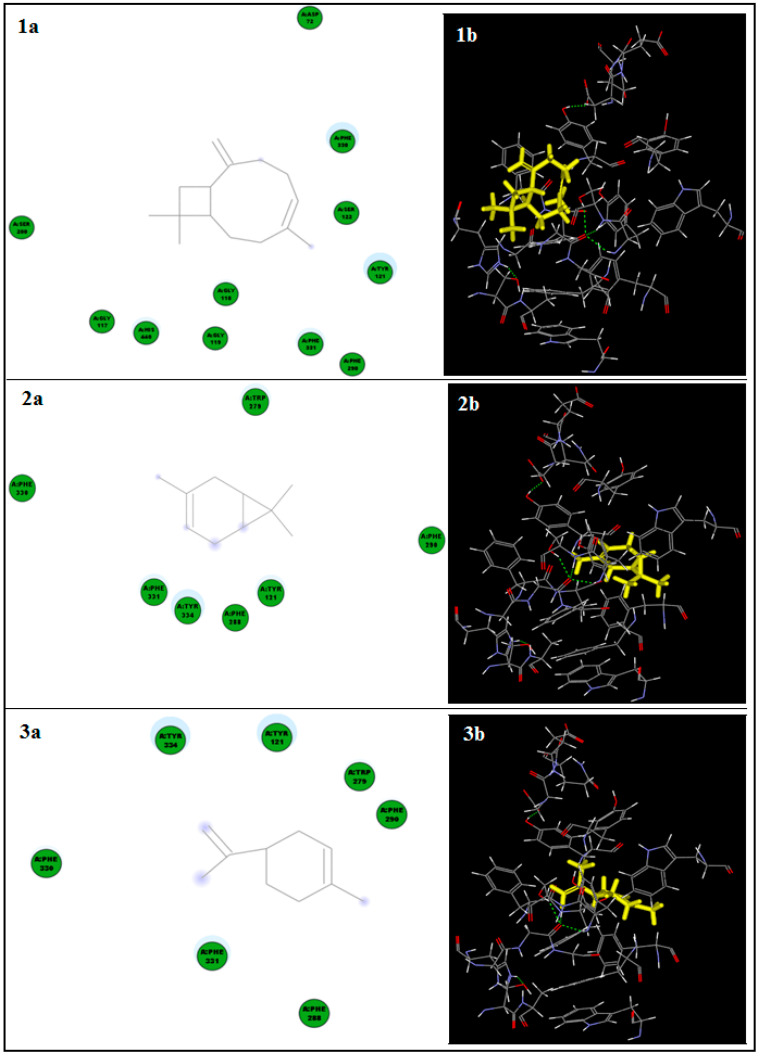
2D-Binding (**a**) and 3D-binding (**b**) diagrams of β-caryophyllene (**1**), 3-carene (**2**), and limonene (**3**) to AChE crystal structure.

In silico molecular docking of each of the identified components (3-carene, limonene, β-caryophyllene, piperine, and sesamin) of black pepper CPO to acetylcholinesterase (AChE) crystal structure was performed. Many amino acid residues were detected in the enzyme active site; among them, seven residues (Phe 288, Phe 290, Phe 330, Phe 331, Tyr 121, Tyr 334, and Trp 279) were determined to stabilize the complex with all the tested ligands. In addition, the residues Asp 72 and His 440 were common in stabilizing the enzyme complex with β-caryophyllene and piperine or sesamin, respectively. The 2D- and 3D-binding diagrams of the docked compounds to AChE crystal structure are presented in Figure 5 and Figure 6. Piperine showed the highest binding affinity, as indicated by its recorded C-Docker binding energy (−7.01 Kcal/mol), H-bond formation with the Phe 288 residue, and hydrophobic interactions with different amino acid residues of the enzyme.

On the other hand, sesamin showed good binding affinity to AChE active site through two Pi–Pi interactions with Tyr 334 and His 440 residues, in addition to hydrophobic interactions with other AChE amino acid residues with a binding energy of 25.80 Kcal/mol. Figure 5E demonstrates the 3D-binding diagram of both piperine and sesamin (the CPO major compounds) to the AChE active site. Due to the hydrophobic nature and low functionality of 3-carene, limonene, and β-caryophyllene, they only showed hydrophobic interactions with the enzyme residues (Figure 6). 

## 4. Discussion

The CPO of black pepper (*Piper nigrum*) fruits was analyzed and biologically assessed for the first time in this study. Both the HDO and CPO of black pepper were comparatively analyzed by GC–MS. HDO oil components showed the presence of higher ratios of monoterpene than sesquiterpene hydrocarbons, which agreed with other published data [34], indicating the elevated oil quality [35]. On the other hand, the CPO contained the lignan sesamin and the alkaloid piperine in high percentages; each constituting more than a third of the oil composition. The presence of sesamin in various *Piper* species has been previously reported, as identified by HPTLC–MS analysis of the extracts of *P. nigrum* and *P. longum* [36]. The presence of limonene, β-caryophyllene, and 3-carene was common in both HDO and CPO. Chen et al. (2020) reported that 3-carene could be used as a quality marker of black pepper oil for evaluating its inhibitory activity against acetylcholinesterase enzyme [37]. Thus, the abovementioned compounds may be potential markers in the quality control analysis of pepper oil. 

The neuroprotective and cognitive-enhancing abilities of *P. nigrum* CPO were investigated against scopolamine-induced memory impairment in rats, and compared to the reference drug donepezil. The results were supported by evaluation of the behavioral and neurochemical parameters, in addition to histopathological and molecular docking studies. 

Measurements of the catalase and MDA levels were used to assess the antioxidant potential of CPO. Catalases break down hydrogen peroxides and reduce the brain oxidative stress by scavenging reactive hydroxyl radicals [38], while malondialdehyde is the lipid peroxidation end product [39]. It is reported that cholinergic function is inversely affected by malondialdehyde levels [40], which further supported our results. 

The effects of the CPO on the cholinergic neurotransmission were evaluated by measuring acetylcholine esterase (AChE) activity. The results of the current study revealed that rats treated with CPO showed a significant decrease in the scopolamine-induced elevation of AChE (51.30%, *p* < 0.05) and restored the normal level of AChE, with a decrease of 3.85% as compared to the control group. Generally, the inhibition of this enzyme leads to the presence of sufficient acetylcholine requirements for proper cognition, learning abilities, and memory retention in the brain [41]. CPO treatment was shown to modify the cholinergic neurotransmission via decreasing the elevated AChE induced by scopolamine into activity levels comparable to donepezil, the used reference drug. Molecular docking results confirmed the AChE inhibitory activity, as seen by the highest binding affinity of piperine to AChE binding site, to which the activity may be mainly attributed. The neuroprotective results were further supported by the histopathological studies of the rats’ brain tissues, which confirmed the absence of alterations in the histological structures and restoration of normal neuronal tissue architecture in the rats receiving CPO.

Since black pepper CPO is a complex mixture of many components, the activity may be related to the synergistic effect of all the constituents. The presence of methylenedioxy groups in both piperine and sesamin or the presence of exocyclic methylene groups in β-caryophyllene allows covalently binding to the amino and sulfhydryl groups of proteins. This interaction causes a dramatic change in the topography of the enzymes and thus loss of activity occurs [42].

However, the activity could be explained based on the individual components as well. Most of the identified components were reported to have antioxidant and neuroprotective effects. Piperine showed potential in vitro antioxidant activity via acting as scavenger of reactive oxygen, superoxide, and hydroxyl radicals, as well as inhibiting the peroxidation of lipids (IC_50_ = 1.23 mM) [17]. Moreover, the administration of piperine to rats subjected to oxidative stress normalized the levels of antioxidant enzymes, such as catalase, reduced glutathione, superoxide dismutase, in addition to glutathione-*S*-transferase and peroxidase [14]. Several studies have reported the neuroprotective activity of piperine occurs through exerting anti-inflammatory and antioxidant effects. Piperine exerted neuroprotection through the inhibition of MPTP-induced toxicity, decreased neuronal inflammation by decreasing the cytokine IL-1β levels, and altered oxidative stress by elevated SOD levels while diminishing lipid peroxidation expression, in addition to enhancing cognitive and motor abilities [43]. Besides, piperine increased the antioxidant parameters through reduction of the glutathione level, inhibition of catalase and COX-2, thus exerting neuroprotection against pilocarpine-induced epilepsy in rats [44]. Moreover, *P. nigrum* extract exerted neuroprotection in the aluminum chloride-induced model of Alzheimer’s via its antioxidant effect, AChE inhibition, and the prevention of neuronal degeneration, tau-protein oligomerization, and amyloid plaque formation [12]. Similar results were obtained in Aβ-induced oxidative stress and memory impairment in the amygdala and hippocampus of rats [11].

Sesamin, the major lignan component, was classified by Kiso (2004) as a pro-antioxidant since its metabolites demonstrated antioxidative roles when sesamin was given orally to rats. They showed potential scavenging activities for both hydroxyl and superoxide radicals [45]. Sesamin was also reported to elevate mice hepatic glutathione peroxidase and reduced glutathione levels, while it decreased the malondialdehyde equivalents and the production of NO and IL-1. Thus, it exerted promising antioxidant, anti-aging, and immunomodulatory effects and improved the brain functions [46]. The neuroprotective effects of sesamin on the Parkinson’s disease model of 1-methyl-4-phenyl-1, 2, 3, 6- tetrahydropyridine (MPTP)-lesioned mice were also assessed. Sesamin ameliorated latency in the passive avoidance test and protected against deficits in habit learning and spatial memory [47]. Moreover, Zhao et al. (2016) reported the ameliorative effects of sesamin on habit-learning and spatial memory deficits in mice subjected to chronic stress induced by chronic electric foot shocks [48], while Baluchnejadmojarad et al. (2017) reported the neuroprotective effects of sesamin in a Parkinson’s model induced by 6-hydroxydopamine, where sesamin decreased the levels of reactive oxygen species, malondialdehyde equivalents in the striatum, and ameliorated the superoxide dismutase activity [49].

β-Caryophyllene demonstrated greater lipid peroxidation inhibitory activity than α-tocopherol, and its antioxidant capacity was evaluated in vitro using the DPPH (IC50 = 1.25 µM) and FRAP (IC50 = 3.23 µM) assays [50,51]. Yang et al. (2017) reported that β-caryophyllene exerted a neuroprotective effect against cerebral injury and ischemia in mice via inhibiting the necroptotic neuronal cell death and reducing the secretion of brain pro-inflammatory cytokines [52]. These results were in agreement with previously published data about β-caryophyllene, showing that it decreased the inflammatory cytokine levels, prevented dopaminergic neuron loss and glial activation, and offered neuroprotection in a murine model of parkinsonism induced by MPTP (1-methyl-4-phenyl-1, 2, 3, 6-tetrahydropyridine) [53]. Similar results demonstrated that β-caryophyllene modulated many inflammatory markers via the inhibition of the expression of interleukin, cyclooxygenase-2, and inducible nitric oxide synthetase, in addition to lowering prostaglandin-E2 and nitric oxide levels [54].

3-Carene was reported to demonstrate good antioxidant and anticholinesterase activities with IC50 values of 0.603 and 0.0358 mg/mL, respectively (Aazza et al., 2011) [55]. These results came following Emami et al. (2013), who evaluated its antioxidative potential using various TLC screening methods, as well as the DPPH assay and non-enzymatic tests of lipid peroxidation [56], while Miyazawa and Yamafuji (2005) reported the potent AChE inhibitory activity of 3-carene [57].

## 5. Conclusions

The neuroprotective activity of black pepper cold-pressed oil was validated via behavioral, neurochemical, and histopathological evaluations. Although the main components of the cold-pressed oil were sesamin and piperine, the presence of limonene, β-caryophyllene, and 3-carene in the oil prepared by two methods (hydrodistillation and cold expression) suggested they were possible markers for the quality control analysis of pepper oil. The in silico molecular docking study confirmed the neuroprotection, showing the highest binding affinity of piperine to the AChE crystal structure, to which activity may be mainly attributed, in addition to fitting of all the other oil components.

## Figures and Tables

**Table 1:** Chemical composition of black pepper hydrodistilled (HDO) and cold-pressed (CPO) oils.

No.	Compound ^a^	RI Calculated	RI Reported	% Composition		Method of Identification
				HDO	CPO	
1.	α-Thujene	918	922	1.31	nd	RI, MS
2.	α-Pinene	925	925	7.27	nd	RI, MS
3.	Camphene	941	943	0.01	nd	RI, MS
**4.**	Sabinene	971	975	21.36	nd	RI, MS
**5.**	β-Pinene	974	979	15.53	nd	RI, MS
6.	β-Myrcene	988	992	2.08	nd	RI, MS
7.	α-Phellandrene	1002	1007	0.36	nd	RI, MS
8.	3-Carene	1008	1011	0.77	9.53	RI, MS
9.	2-Carene	1015	1014	0.43	nd	RI, MS
10.	*p*-Cymene	1023	1025	0.07	nd	RI, MS
**11.**	Limonene	1029	1032	26.50	6.23	RI, MS
12.	Eucalyptol	1031	1031	0.07	nd	RI, MS
13.	*cis*-β-Ocimene	1037	1037	0.02	nd	RI, MS
14.	*trans*-β-Ocimene	1047	1050	0.15	nd	RI, MS
15.	γ-Terpinene	1058	1060	1.15	nd	RI, MS
16.	*cis*-Sabinene hydrate	1067	1067	0.24	nd	RI, MS
17.	Terpinolene	1087	1089	0.27	nd	RI, MS
18.	β-Linalool	1100	1100	0.69	nd	RI, MS
19.	Terpinene-4-ol	1178	1177	0.59	nd	RI, MS
20.	α-Terpineol	1191	1190	0.02	nd	RI, MS
21.	δ-EIemene	1340	1337	0.07	nd	RI, MS
22.	α-Copaene	1379	1377	0.17	nd	RI, MS
23.	*cis*-α-Bergamotene	1418	1415	0.10	nd	RI, MS
**24.**	β-Caryophyllene	1427	1428	19.12	10.67	RI, MS
25.	*trans*-α-Bergamotene	1438	1435	0.18	nd	RI, MS
26.	(E)-β-Famesene	1457	1456	0.06	nd	RI, MS
27.	α-Humulene	1460	1453	0.10	nd	RI, MS
28.	Isocaryophyllene	1489	1494	0.02	nd	RI, MS
29.	α-Selinene	1493	1493	0.02	nd	RI, MS
30.	α-Zingiberene	1499	1495	0.14	nd	RI, MS
31.	Valencene	1502	1496	0.02	nd	RI, MS
32.	β-Bisabolene	1512	1508	0.33	nd	RI, MS
33.	δ-Cadinene	1529	1523	0.07	nd	RI, MS
34.	α-Farnesene	1536	1538	0.19	nd	RI, MS
35.	Caryophyllene oxide	1591	1593	0.16	nd	RI, MS
36.	Piperine	2915	2933	nd	33.79	RI, MS, (SI = 94)
37.	Sesamin	3145	3151	nd	39.78	RI, MS, (SI = 92)
	Total			99.64%	100%	

^a^ Compounds are numbered based on their order of elution on Rtx-5MS fused-bonded column; MS: mass spectrum; nd: not detected; RI: retention index; SI: similarity index, which is a factor for directly matching the spectrum of the unknown sample and that of the library; major components are highlighted in bold.

## Data Availability

Data is available within the article and supplementary materials.

## Supplementary Materials

The following are available online at https://www.mdpi.com/article/10.3390/antiox10121993/s1, Figure S1: GC chromatogram of cold-pressed oil (CPO) of black pepper fruits, Figure S2: GC chromatogram of hydrodistilled oil (HDO) of black pepper fruits.
