# Supplementary material for: Neuroprotective Effects of Black Pepper Cold-Pressed Oil on Scopolamine-Induced Oxidative Stress and Memory Impairment in Rats
Source: Antioxidants (Basel). 2021 Dec 15;10(12):1993. doi: 10.3390/antiox10121993 (PMC8698347; doi:10.3390/antiox10121993)
Supplement: Supplementary file 1 [file antioxidants-10-01993-s001.zip › antioxidants-1464492-supplementary.pdf]

Supplementary material

Article

Neuroprotective effects of black pepper cold-pressed oil on scopolamine-induced oxidative stress and memory impairment in rats

Nada M. Mostafa[1,*], Ahmed M. Mostafa[2], Mohamed L. Ashour [1,3,*] and Sameh S. Elhady [1]

1. Department of Pharmacognosy, Faculty of Pharmacy, Ain-Shams University, Cairo 11566, Egypt
2. Department of Biochemistry, Faculty of Pharmacy, Ain-Shams University, Cairo 11566, Egypt; ahmedmostafa@pharma.asu.edu.eg
3. Department of Pharmaceutical Sciences, Pharmacy Program, Batterjee Medical College, Jeddah 21442, Saudi Arabia
4. Department of Natural Products, Faculty of Pharmacy, King Abdulaziz University, Jeddah 21589, Saudi Arabia; ssahmed@kau.edu.sa

* Correspondance: nadamostafa@pharma.asu.edu.eg (N.M.M.) ; ashour@pharma.asu.edu.eg (M.L.A.)

Abstract: Oxidative stress is usually associated with many neurodegenerative diseases. In this study, the gas chromatography–mass spectrometry (GC–MS) analysis of cold-pressed oil (CPO) from black pepper (*Piper nigrum*) fruits was performed and its neuroprotective effects were evaluated for the first time. The analysis of CPO revealed the presence of the lignan sesamin (39.78%), the alkaloid piperine (33.79%), the monoterpene hydrocarbons 3-carene (9.53%) and limonene (6.23%), and the sesquiterpene β-caryophyllene (10.67%). Black pepper hydrodistilled oil (HDO) was also comparatively analyzed by GC–MS to show the impact of oil isolation by two different methodologies on their components and class of compounds identified. HDO analysis revealed 35 compounds (99.64% of the total peak areas) mainly composed of monoterpene hydrocarbons (77.28%), such as limonene (26.50%), sabinene (21.36%), and β-pinene (15.53%), and sesquiterpene hydrocarbons (20.59%) represented mainly by β-caryophyllene (19.12%). Due to the low yield obtained for HDO (0.01% *v/w*), only CPO was chosen for the evaluation of its neuroprotective potential. Alzheimer-type dementia was induced in rats by scopolamine intraperitoneal injection (1.5 mg/kg/day) for seven days. CPO was administered orally (100 mg/kg) for a week before scopolamine administration and then concomitantly for another week. Donepezil (1 mg/kg, orally) was used as a reference drug. CPO administration significantly improved the rat behaviors as evaluated by the Morris water maze test, evident from prolongation in time spent in the platform quadrant (262.9%, compared to scopolamine) and increasing in the crossing time by 18.18% compared to the control group. The rat behavior tested by passive avoidance, showed prolongation in the step-through latency compared to control. Moreover, CPO significantly ($p < 0.05$) ameliorated the activities of antioxidant enzymes such as catalase, superoxide dismutase (SOD) and reduced malondialdehyde (MDA) equivalents by 22.48%, 45.41%, and 86.61%, respectively, compared to scopolamine. Furthermore, CPO administration decreased scopolamine-induced elevated acetylcholinesterase levels in rats' hippocampi by 51.30%. These results were supported by histopathological and in silico molecular docking studies. Black pepper oil may be a potential antioxidant and neuroprotective supplement.

Keywords: Black pepper; cold-pressed oil; GC/MS; neuroprotective; scopolamine.

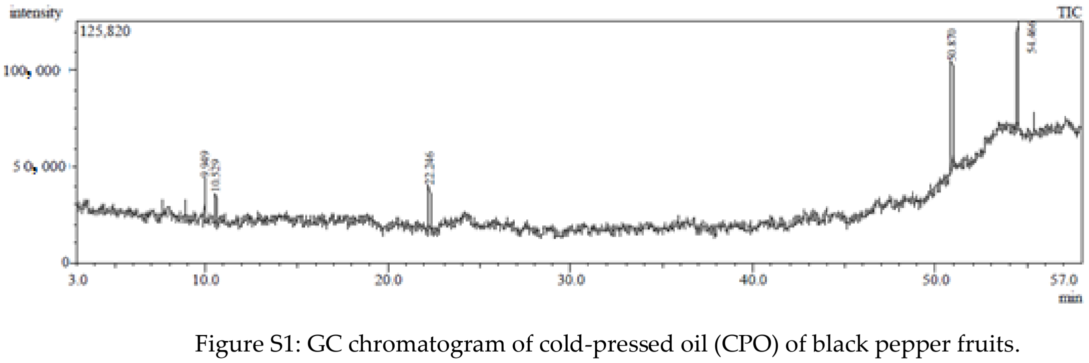

Figure S1: GC chromatogram of cold-pressed oil (CPO) of black pepper fruits.

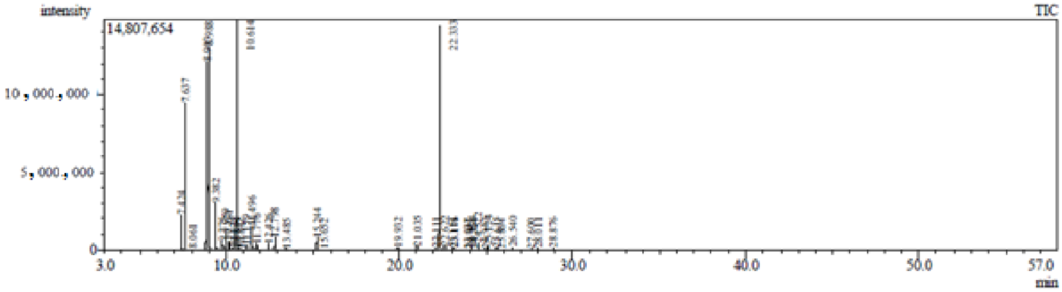

Figure S2: GC chromatogram of hydrodistilled oil (HDO) of black pepper fruits.
